# Analysis of Subdural Injection During Lumbar Interlaminar Epidural Injection in Failed Back Surgery Syndrome

**DOI:** 10.3390/jcm9103132

**Published:** 2020-09-28

**Authors:** Jin Young Lee, Woo Seog Sim, Ji Yeong Kim, Yu Ri Ko, So Young Lee, Mihyeon Lee, Seunghee Cho, Hue Jung Park

**Affiliations:** 1 Department of Anesthesiology and Pain Medicine, Samsung Medical Center, School of Medicine, Sungkyunkwan University, Seoul 06351, Korea; L7035@hanmail.net (J.Y.L.); wooseog.sim@samsung.com (W.S.S.); 2 Department of Anesthesiology and Pain Medicine, Seoul St. Mary’s Hospital, College of Medicine, The Catholic University of Korea, Seoul 06591, Korea; apple_queen@naver.com (J.Y.K.); finocchio@daum.net (Y.R.K.); slamfor01@gmail.com (S.Y.L.); mihyun2023@naver.com (M.L.); 3 Department of Anesthesiology and Pain Medicine, Incheon St. Mary’s Hospital, College of Medicine, The Catholic University of Korea, Incheon 21431, Korea; soksakim@naver.com

**Keywords:** epidural, interlaminar, lumbar, subdural, failed back surgery syndrome

## Abstract

Persistent or recurrent back and leg pain following spinal surgery, known as failed back surgery syndrome (FBSS), significantly limits daily life activities. A lumbar epidural injection can reduce adhesions, inflammation, and nerve compression, although the epidural space can be distorted due to dura mater and epidural tissues changes after spinal surgery. This study analyzed subdural injection during lumbar epidural injection in FBSS patients. We retrospectively analyzed data from 155 patients who received a lumbar interlaminar epidural injection to manage FBSS. We grouped the patients based on the injected contrast medium appearance in the subdural (group S) or epidural spaces (group E) in fluoroscopic contrast images. Demographic, clinical, surgical and fluoroscopic data were recorded and evaluated, as were the pain scores before and after injection. There were 59 patients (38.1%) in the subdural group. Injection distance from the surgery level differed between the groups. Risk of subdural injection at level 1 distance from the surgery level had an odds ratio of 0.374, and at level ≥2, it was 0.172, when compared to level 0. Subdural incidence differed with the distance from surgical site. Physicians should strive to reduce subdural incidence when the injection is planned at surgery site in FBSS.

## 1. Introduction

Persistent and recurrent back and leg pain following spinal surgery significantly limits everyday life activities [1]. Failed back surgery syndrome (FBSS) has many etiologies: including lesions to disc or facet area adjacent to the surgery site; persistent or recurrent neural compression; neuritis; fibrosis; hardware-associated pain; and psychosocial factors [2,3,4,5]. Lumbar epidural injection of local anesthetics, steroids, and hyaluronidase limits adhesions, inflammation, and nerve compression in patients with FBSS [2,6,7,8]. However, the lumbar epidural space can become distorted due to spinal surgery-associated dura mater and epidural tissue changes [9,10,11]. The subdural space is described as a potential cavity between the dura and arachnoid maters [12]. Reina and colleagues [12] examined the ultrastructure of spinal meninges in human cadavers. They reported that the arachnoid mater had two parts: a compact laminar part covering the dural sac’s internal surface, and a trabecular part extending like a spider web around the pia mater of the spinal cord and the medullar roots. The space between the laminar arachnoid and the innermost dura mater layer is known as the dura-arachnoid interface [12]. A subdural space can be formed by the junctions between the neurothelial cells breaking due to mechanical pressure, air, or injected fluid, creating fissures within the interface [12]. These fissures grow larger towards weaker areas, producing an incomplete form, or creating an actual subdural space [12]. The formation of a subdural space by post-surgical changes in patients with FBSS has not been evaluated. If the subdural space forms after surgery, incidence of injections into subdural space may increase, even when using a routine, standardized epidural injection technique. Moreover, uncertain tactile feedback when using the loss of resistance (LOR) technique can increase subdural injection risk due to epidural tissue changes. Accidental subdural injection of local anesthetics can lead to devastating neurological complications, including high sensory and motor block, moderate hypotension, bradycardia, progressive respiratory difficulty, loss of consciousness, or cardiac arrest [13,14,15]. The subdural incidence is reported to be between 0.03% and 3.2% in epidural blocks [12,13,15,16]. However, subdural injection mechanisms, incidence, and patterns among patients with FBSS are not known. In this study, we analyzed subdural injection to identify the differences in subdural incidence based on the injection distance from the surgery level among patients with FBSS.

## 2. Materials and Methods

### 2.1. Patients

We retrospectively reviewed the electronic medical records of 157 patients with lower back and leg pain in FBSS, who underwent lumbar interlaminar epidural injection between July 2019 and March 2020 at two tertiary care hospitals. The patients’ ages ranged from 29 to 90 years old. The inclusion criteria were: (a) over six months had elapsed since the patients’ lumbar spinal surgery; (b) a primary diagnosis of lower back pain radiating to the lower limbs; (c) a cross-sectional imaging study (either computed tomography or magnetic resonance imaging) of the lumbosacral spine among patients diagnosed with spinal stenosis or herniated nucleus pulposus [17]. The exclusion criteria included lumbar vertebroplasty, lumbar neuroplasty, neoplastic diseases, peripheral vascular disease, or the use of medications affecting the vascular system [17]. The epidural injection level was chosen based on clinical manifestations, physical examination, and review of the imaging studies [17]. Lesion severity was categorized as one of three levels (mild, moderate, severe) by reviewing the imaging data [17]. This study was approved by our departmental ethics committee (KC20RIDI0315, SMC 2020-03-129) and registered with CRIS (Clinical Research Information Service of the Korea National Institute of Health, http://cris.nih.go.kr/cris/index.jsp, KCT0004892). The need for individual consent was waived by the institutional review board, as this was a retrospective study involving medical record review.

### 2.2. Interventions

All procedures were standardized and performed by one of six experienced pain physicians under fluoroscopic guidance. Patients were placed in the prone position, and anteroposterior and lateral view images were obtained with a C-arm (OEC series 9800, GE healthcare, Chicago, IL, USA) to ensure the proper site of entry. Following aseptic preparation and application of 1% lidocaine, a 20-gauge Tuohy needle (Tae-Chang Industrial Co., Seoul, Korea) was inserted through the skin surface and into the interlaminar space of the target spinal lesion. Aspirations to assess for the presence of blood or cerebrospinal fluid were routinely performed. When LOR was felt, the aspiration test was performed, then 3–5 mL of contrast medium (Omnipaque^®^, 300mgI.ml^−1^, GE Healthcare, Little Chalfont, Buckinghamshire, UK) was injected to confirm the needle position in the epidural space. The contrast medium spread pattern was defined as a subdural or epidural spread. We assumed subdural spread when the contrast medium was observed to be thick, loculation (elongated high density) away from the posterior epidural space in lateral view, and not extending or outlining to the foramen in anteroposterior view [14]. When subdural contrast spread was observed, the procedure was terminated, and psoas compartment injection was performed with 10 mL 0.4% lidocaine on the affected side. In the case of subdural injection during a midline interlaminar approach, psoas injection was performed on both sides. We assumed epidural spread when the contrast medium was observed between the anterior and posterior epidural space in lateral and anteroposterior views. After confirming epidural space positioning, an injectate volume of 7 mL, containing 0.4% lidocaine, dexamethasone 5 mg, and hyaluronidase 750 IU, was infused. Injection distance from the surgical site was defined by intervertebral levels. When the injection levels were within the surgical level, it was defined as level 0. When the injection was 1 or ≥2 intervertebral levels away from the surgical level, it was defined as level 1 or level ≥ 2, respectively. Two physicians participating in the procedure analyzed the fluoroscopic images. Following the procedure, the patients were observed for any adverse effects. The pain was scored using a numerical rate scale (NRS, ranging from 0 = no pain to 10 = absolutely intolerable pain). The pain score was recorded before and 30 min after the epidural or psoas compartment injection.

### 2.3. Statistical Analysis

All data were analyzed using SAS 9.4 (SAS Institute, Cary, NC, USA). Data are expressed as the mean ± standard deviation (SD) or numbers (percentages), as appropriate. We assigned patients to group S when subdural contrast medium was observed after the injection, and into group E when epidural contrast medium was observed after the injection. When subdural and epidural contrast medium were simultaneously demonstrated, the patient was categorized into group S. Demographic data for the two groups were compared using a Wilcoxon rank sum test, Chi-square test, *t*-test or Fisher’s exact test. Comparisons for pain severity between groups, and before and after block in each group were performed using the Wilcoxon rank sum test. Logistic regression analysis was used to predict the distance factor between the occurrence of subdural injection and injection distance from the surgical site. A *p*-value less than 0.05 was considered statistically significant.

## 3. Results

Of the 157 patients deemed eligible, two patients for whom the injection was at level 1 were excluded because the failed epidural injection was associated with epidural venogram during epidural approach, at which point the procedure was halted with no complications. Thus, data of 155 patients were analyzed. Demographic and clinical data are summarized in Table 1. Age, sex, body mass index, diagnosis, duration of pain, lesion level, lesion severity, and sensory and motor disturbance of leg did not differ between the two groups (Table 1). Surgery type, time after spinal surgery, number of spinal surgery, and surgery range also did not differ between the two groups (Table 2). In the fluoroscopic data, the injection side and attempt number were similar between the groups (Table 3). The injection level differed between the groups. The most common injection level was L4–5. The proportions of all other injection levels were lower in group S. The injection distance from surgery differed between the groups (*p* < 0.001). The post hoc power was 99.5%. The subdural incidence varied with the injection distance from the surgical site (level 0 vs. level 1 vs. level ≥ 2, *p* = 0.001). The subdural incidence differed between level 1 vs. level 0 (odds ratio 0.374, 95% CI 0.163–0.860, *p* = 0.016) and in level ≥ 2 vs. level 0 (odds ratio 0.172, 95% CI 0.050–0.594, *p* = 0.003) (Table 4). The pain score before or after injection was not different between the groups. The pain score was significantly lower after the injection among both groups (*p* < 0.001). Among group S, headache (*n* = 3), chest tightness (*n* = 1), and dizziness (*n* = 1) were observed after injection and were improved. All patients were monitored approximately 30 min to 1 h after the procedure. After disappearance of these symptoms, confirmation of stable vital signs, and sensory and motor examinations of extremities, patients were discharged. None of the cases displayed any evidence of dural puncture, hemodynamic instability, or unexpected high sensory and motor block.

## 4. Discussion

In the present study, we aimed to analyze subdural spread during lumbar interlaminar epidural injection in FBSS patients. We observed that the rate of subdural incidence was 38.1%. This was considerably higher than the previously reported 0.82% of subdural incidence in epidural blocks, which was based on non-back surgery and back surgery patients, with diagnostic methods using subdural clinical findings, not fluoroscopic findings [15]. The subdural incidence differed according to the distance between the injection and surgical levels. Level ≥ 2 distance from the surgical level displayed a lower odds ratio than level 1 distance. We suspect that a larger distance from the surgical level reduces the risk for subdural injection, possibly by avoiding the post-surgical fibrosis and adhesions. Group S involved lower proportions than group E at all other levels except L4–5 level. This difference in proportions might be related to the subdural incidence risk in association with the distance from the spinal lesion. Such lesions are mostly reported at L4–5 and L5–S1 levels. However, we did not evaluate the association between each of the injection levels and subdural incidence. Group S underwent psoas compartment injection, yet there was no difference in the pain score between the two groups after injection. Psoas compartment injection might provide efficient analgesia, and possibly acts as an epidural injection. However, we used different injectate mixture (only lidocaine) and volume (10 mL) in psoas compartment injections. Therefore, further expanded trials are needed to evaluate the analgesic efficacy between these two treatments.

FBSS is characterized by persistent, chronic, and disabling pain following spine surgery [9,18]. Epidural and perineural fibrosis are major contributing factors for lower back and radicular pain, accounting for up to 36% of FBSS [9,10,11,19,20]. The formation of epidural fibrosis is an inevitable complication of laminectomy [9]. The possible mechanism includes the migration of lymphocytes, fibroblasts, and macrophages from paravertebral tissue into the surgical site. The migrating cells contribute to the progression of peridural fibrosis [9,19,21]. Spinal epidural fibrosis and adhesions might cause dural mater compression or peridural tethering. The compression and tethering might cause pain, and the fibrosis and adhesions might prevent injectate delivery into the epidural space [9,18]. Epidural fibrosis has been reported in animal laminectomy models [22,23]. The presence of fibrosis increases the re-operation time and the risk of dural tears [22]. Ozturk and colleagues [19] described an arachnoid membrane becoming thickened and adherent to the dura mater after total lumbar laminectomy in a rat model. In FBSS, the main reason for epidural injection failure is surgery-induced perineural fibrosis that impedes injectate delivery to the target lesion [20]. We suspect that epidural and dura mater changes following surgery also cause difficulties during epidural approach before epidural injection.

Epidural injections are commonly utilized interventions in FBSS [6,7,24,25,26]. Inaccurate needle placement leads to injectate failure to the target level and accidental subdural, intradural, subarachnoid, or intravascular injection [7]. Subdural injection is defined by two major criteria; a negative aspiration test, and an unexpected widespread sensory block, and three minor criteria; delayed onset, variable motor block, and extensive sympatholysis [15,27]. The presence of one major criteria and at least one minor criteria is defined as being a highly diagnostic sign of subdural injection [15,27]. The subdural injection can rapidly affect multiple spinal levels. When the local anesthetics reach the brain stem, they lead to apnea and unconsciousness [14]. Several cases of subdural injection, but no controlled studies, have been reported [13,14,16,28,29]. Lubenow and colleagues [15] reported subdural incidence in 18 out of 2182 patients (0.82%) treated by lumbar epidural injection. Only five of the 18 patients (27.8%) had previously had back surgery. These researchers suggested that back surgery patients are prone to accidental subdural injection because the epidural anatomy may have changed due to scarring and retraction. These changes result in a narrow epidural and a wide subdural space [15]. Classic risk factors for subdural injection include previous back surgery, recent lumbar puncture, and difficult block placement, but the evidence for these comes from single case reports or data on a small number of patients [27,30,31]. Our study shows a higher subdural incidence than in a previous report on epidural block [15]. The injection distance from the surgical site was categorized with level 0, 1, or ≥2 away from the surgery level. The level ≥ 2 displayed the lowest odds ratio for subdural incidence. New lesions in FBSS might occur near the surgery level [26,32]. Recurrent disc herniation following discectomy might occur at the same or adjacent level [33]. In group S, the proportion of patients at level ≥ 2 was smaller than those at level 0 and 1. However, these results are highly reliable, as our data had a statistical power of 99.5%. Further studies with a large sample size are required to ascertain the correlation between the distance from the surgical level and the subdural incidence.

This study has several limitations. Firstly, we did not record the presence and/or grade of tactile feed-back when using the LOR technique. Even experienced physicians cannot detect the exact LOR feed-back in distorted or narrowed epidural space. Secondly, we enrolled laminectomy patients, who lacked the ligamentum flavum after the surgery, so the tactile feedback during LOR was minimal or absent. This could contribute to increased subdural incidence. Thirdly, we did not subdivide the distance of level 2 or more from the surgical level. Rather, we included all these patients into a single category as level ≥ 2. Finally, 30 min after the injection was rather a short time for analgesic efficacy evaluation.

## 5. Conclusions

We observed a subdural incidence of 38.1% during lumbar interlaminar epidural injection among patients with FBSS. Injection distance from the surgical level differed with subdural incidence. Physicians should determine the injection level considering the patient’s clinical symptoms and possibility of subdural incidence in FBSS. Physicians should also make every attempt to reduce subdural incidence when an interlaminar epidural injection is planned at a level close to the surgical level among patients with FBSS. Moreover, closer monitoring to detect subdural injection is necessary during the procedure. Further controlled trials are needed to identify subdural incidence and patterns in different types of epidural injection and at various distances from the surgical level among patients with FBSS.

## Figures and Tables

**Table 1:** Demographic and clinical characteristics.

	All Patients (*n* = 155)	Group S (*n* = 59)	Group E (*n* = 96)	*p*-Value
Age (y)	65.9 ± 11.9	65.9 ± 11.7	66.0 ± 12.1	0.985
Sex (M/F)	67/88	23/36	44/52	0.403
Body mass index (kg/m^2^)	24.4 ± 2.8	24.5 ± 2.8	24.2 ± 2.9	0.603
Dignosis Spinal stenosis HNP	105 (67.7%) 50 (32.3%)	41 (69.5%) 18 (30.5%)	64 (66.7%) 32 (33.3%)	0.715
Duration of pain (y) <1 1–2 >2	22 (14.2%) 15 (9.7%) 118 (76.1%)	5 (8.5%) 4 (6.8%) 50 (84.8%)	17 (17.7%) 11 (11.5%) 68 (70.8%)	0.139
Lesion level L1–2 L2–3 L3–4 L4–5 L5–S1	8 (5.2%) 21 (13.6%) 31 (20.0%) 65 (41.9%) 30 (19.4%)	3 (5.1%) 11 (18.8%) 29 (49.2%) 8 (13.6%) 8 (13.6%)	5 (5.2%) 19 (19.8%) 36 (37.5%) 23 (24.0%) 13 (13.5%)	0.527
Lesion severity Mild Moderate Severe	12 (7.7%) 92 (59.4%) 51 (32.9%)	1 (1.7%) 39 (66.1%) 19 (32.2%)	11 (11.5%) 53 (55.2%) 32 (33.3%)	0.072
Sensory disturbance of leg Normal Mild Moderate Severe	100 (64.5%) 27 (17.4%) 18 (11.6%) 10 (6.5%)	37 (62.7%) 11 (18.6%) 6 (10.2%) 5 (8.5%)	63 (65.6%) 16 (16.7%) 12 (12.5%) 5 (5.2%)	0.824
Motor disturbance of leg Normal Mild Moderate Severe	88 (56.8%) 31 (20.0%) 22 (14.2%) 14 (9.0%)	55 (57.3%) 17 (17.7%) 16 (16.7%) 8 (8.3%)	33 (55.9%) 14 (23.7%) 6 (10.2%) 6 (10.2%)	0.594

All data are presented as the mean ± SDs or number (%) of patients. HNP: herniated nucleus pulposus, Group S: patients who showed subdural contrast injection, Group E: patients who showed epidural contrast injection; *p*-value < 0.05 was considered statistically significant.

**Table 2:** Surgical characteristics.

	All Patients (*n* = 155)	Group S (*n* = 59)	Group E (*n* = 96)	*p*-Value
Surgery type PLIF Laminectomy Discectomy	76 (49.0%) 45 (29.0%) 34 (21.9%)	30 (50.9%) 15 (25.4%) 14 (23.7%)	46 (47.9%) 30 (31.3%) 20 (20.8%)	0.729
Time after surgery ≤2 year >2 year	38 (24.5%) 117 (75.5%)	13 (22.0%) 46 (78.0%)	25 (26.0%) 71 (74.0%)	0.573
Number of spinal surgery 1 2 ≥3	107 (69.0%) 32 (20.7%) 16 (10.3%)	40 (67.8%) 12 (20.3%) 7 (11.9%)	67 (69.8%) 20 (20.8%) 9 (9.4%)	0.885
Surgery range (level) 1 2 3 4 5	110 (71.0%) 30 (19.4%) 9 (5.8%) 3 (1.9%) 3 (1.9%)	39 (66.1%) 11 (18.6%) 5 (8.5%) 2 (3.4%) 2 (3.4%)	71 (74.0%) 19 (19.8%) 4 (4.2%) 1 (1.0%) 1 (1.0%)	0.147

All data are presented as the number (%) of patients. PLIF: posterior lumbar interbody fusion, Group S: patients who showed subdural contrast injection, Group E: patients who showed epidural contrast injection; *p*-value < 0.05 was considered statistically significant.

**Table 3:** Fluoroscopic data and pain severity change.

	All Patients (*n* = 155)	Group S (*n* = 59)	Group E (*n* = 96)	*p*-Value
Lesion level L1–2 L2–3 L3–4 L4–5 L5–S1	8 (5.2%) 14 (9.0%) 20 (12.9%) 70 (45.2%) 43 (27.7%)	1 (1.7%) 4 (6.8%) 4 (6.8%) 35 (59.3%) 15 (25.4%)	7 (7.3%) 10 (10.4%) 16 (16.7%) 35 (36.5%) 28 (29.2%)	0.042
Injection side Left/ Right/ Middle	64/45/46	23/15/21	41/30/25	0.433
Attempt number 1/2/3	139/13/3	54/4/1	85/9/2	0.897
Injection distance from surgery Level 0 Level 1 Level ≥ 2	62 (40.0%) 64 (41.3%) 29 (18.7%)	34 (57.6%) 20 (33.9%) 5 (8.5%)	28 (29.2%) 44 (45.8%) 24 (25.0%)	<0.001
Pain severity (NRS) Before block After block	7.1 ± 1.5 5.3 ± 2.3	7.1 ± 1.3 5.7 ± 1.8	7.1 ± 1.6 5.0 ± 2.5	0.873 0.095

All data are presented as the mean ± SDs or number (%) of patients. NRS: numerical rate scale, Group S: patients who showed subdural contrast injection, Group E: patients who showed epidural contrast injection; *p*-value < 0.05 was considered statistically significant.

**Table 4:** Logistic regression analysis for injection distance from surgical level and subdural incidence.

Injection Distance from Surgical Level	Odds Ratio	95% Confidence Interval		*p*-Value
		Lower Limit	Upper Limit	
Level 1 vs. Level 0 Level ≥ 2 vs. Level 0	0.374 0.172	0.163 0.050	0.860 0.594	0.016 0.003

Level 0; same intervertebral level from surgical level, Level 1; one intervertebral level away from surgical level, Level ≥ 2; over two intervertebral level away from surgical level; *p*-value < 0.05 was considered statistically significant.
